# Cardiac magnetic resonance assessed valve morphology and aortic distensibility in severe aortic stenosis

**DOI:** 10.1186/1532-429X-14-S1-P92

**Published:** 2012-02-01

**Authors:** Christopher D Steadman, Gerry P McCann

**Affiliations:** 1 University of Leicester, Leicester, UK

## Summary

We have shown that cardiac magnetic resonance (CMR) can accurately assess aortic valve morphology in severe aortic stenosis (AS) and this is associated with significant differences in aortic distensibility (AD) despite similar degrees of stenosis severity.

## Background

AD is known to be a prognostic marker in other diseases. The effect of valve morphology on AD has not been reported in patients with severe AS without coronary artery disease.

## Methods

46 patients with severe AS without obstructive coronary artery disease awaiting aortic valve replacement were studied. Transthoracic echocardiography (TTE) was used to assess aortic valve area using the continuity equation, peak aortic velocity and mean valve gradient. CMR was used to assess aortic valve morphology and to enable planimetry of aortic valve area. Two SSFP cines of the aortic valve were taken; one planned from a 3-chamber cine at the level of the aortic annulus in end-diastole and one at the aortic valve tips in end-systole. The smallest planimetered valve area was used for analysis. A high temporal resolution cine of the ascending aorta was taken at the level of the pulmonary artery bifurcation with three blood pressure recordings taken at the same time; the average of the three readings was used for analysis. AD was calculated according to the formula (aortic area max - aortic area min/ aortic area max)*(systolic BP - diastolic BP).

## Results

**Figure 1:**
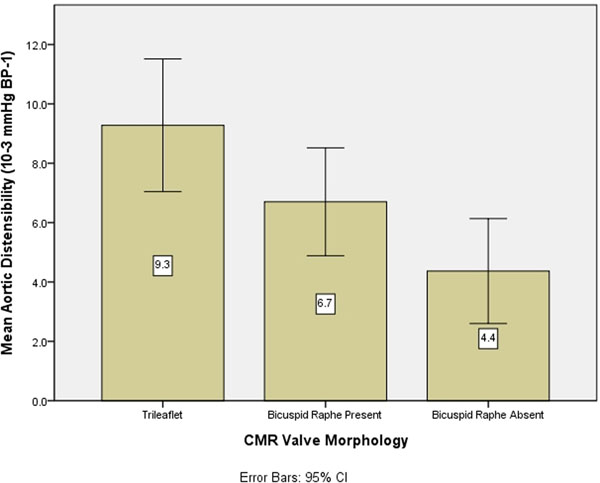
CMR valve morphology and relationship with aortic distensibility.

Baseline characteristics; Males 74%, CMR aortic valve area 0.76±0.21 cm2 TTE aortic valve area 0.86±0.22 cm2, peak velocity 4.4±0.6m/s and mean gradient 48.5±14.1mmHg. AD was not normally distributed therefore was log transformed before analysis. The valve morphology could be clearly seen in all patients. More than half of the patients (63%) studied had bicuspid aortic valves, see Table 1. The bicuspid valves were classified as either Type 1 (fusion of right and left coronary cusps), Type 2 (fusion of right and non-coronary cusps), or Type 3 (fusion of left and non-coronary cusps). Of the 22 bicuspid valves with a raphe 14 were Type 1, eight Type 2 and no Type 3, of the seven without a raphe five were Type 1, two Type 2 and no Type 3. Valve morphology did not significantly correlate with valve area (TTE or CMR) or velocities/gradients. AD was not correlated with sex, age, aortic valve areas (CMR or TTE) or velocities/gradients. AD significantly decreased from trileaflet to bicuspid valve with raphe present and then further to bicuspid valve with raphe absent (ANOVA p=0.01), Figure 1.

**Table 1:** Differences between valve morphologies

	Trileaflet	Bicuspid raphe present	Bicuspid raphe absent	p-value
Number of patients	17	22	7	
Aortic valve area CMR (cm sq)	0.76+-0.24	0.78+-0.20	0.70+-0.15	0.71
Aortic valve area TTE (cm sq)	0.88+-0.27	0.85+-0.21	0.83+-0.15	0.83
Maximum aortic velocity (m/s)	4.4+-0.5	4.6+-0.6	4.1+-0.4	0.12
Mean pressure gradient (mmHg)	46.2+-12.7	53.0+-15.0	39.8+-9.3	0.06
Aortic distensibility (10-3 mmHg BP-1)	9.3+-4.3	6.7+-4.1	4.4+-1.7	0.01*

## Conclusions

CMR is uniquely placed to assess aortic valve morphology and assess vascular function. We have demonstrated that significant numbers of severely stenosed aortic valves are functionally bicuspid and that these valves are associated with impaired vascular function when compared with trileaflet valves. Valves that are truly bicuspid are associated with further decreased AD. The prognostic implications of such findings warrant further study.

## Funding

This study was funded by a Project Grant from the British Heart Foundation. Further support was received from the Leicester Cardiovascular Biomedical Research Unit.

